# Readability of informed consent forms for whole-exome and whole-genome sequencing

**DOI:** 10.1007/s12687-017-0324-6

**Published:** 2017-08-31

**Authors:** Emilia Niemiec, Danya F. Vears, Pascal Borry, Heidi Carmen Howard

**Affiliations:** 1 Erasmus Mundus Joint International Doctoral (Ph.D.) Degree Programme in Law, Science and Technology, University of Bologna, Via Galliera 3, 40121 Bologna, Italy; 2 Department of Law, University of Turin, Lungo Dora Siena 100 A, 10153 Turin, Italy; 3 Centre for Ethics and Law in the Life Sciences, Leibniz University Hannover, Am Klagesmarkt 14-17, 30159 Hannover, Germany; 4 Centre for Biomedical Ethics and Law, Department of Public Health and Primary Care, KU Leuven, Kapucijnenvoer 35, Box 7001, 3000 Leuven, Belgium; 5 Leuven Institute for Human Genomics and Society, 3000 Leuven, Belgium; 6 Centre for Research Ethics and Bioethics, Uppsala University, Box564, SE-751 22 Uppsala, Sweden

**Keywords:** Informed consent, Readability, Whole genome sequencing, Whole exome sequencing, Genetic counselling

## Abstract

Whole-exome and whole-genome sequencing (WES, WGS) can generate an unprecedented amount of complex information, making the informed consent (IC) process challenging. The aim of our study was to assess the readability of English IC forms for clinical whole-exome and whole-genome sequencing using the SMOG and Flesch-Kincaid formulas. We analysed 36 forms, most of which were from US providers. The median readability grade levels were 14.75 (the SMOG formula) and 12.2 (the Flesch-Kincaid formula); these values indicate the years of education after which a person would be able to understand a text studied. All forms studied seem to fail to meet the average recommended readability grade level of 8 (e.g. by Institutional Review Boards of US medical schools) for IC forms, indicating that the content of the forms may not be comprehensible to many patients. The sections aimed at health care professionals (HCPs) in the forms indicate that HCPs should be responsible for explaining IC information to the patients. However, WES and WGS may be increasingly offered by primary care professionals who may not (yet) have sufficient training to be able to communicate effectively with patients about genomics. Therefore, to secure an adequate, truly informed consent process, the task of developing good, legible examples of IC forms along with educating HCPs in genomics should be taken seriously, and adequate resources should be allocated to enable these tasks.

## Introduction

### The challenge of informed consent

Informed consent (IC) was introduced into research practice as an instrument enabling choice about participation in a study, with the aims to prevent coercion and respect autonomy of research participants, mostly in response to research malpractices that occurred in the last century (Hoeyer 2009). The Declaration of Helsinki written in 1964 and amended in subsequent years set the standards for more explicit, documented and specific (i.e. containing a defined set of elements) informed consent in research (World Medical Association 2004). These requirements were gradually implemented both in research and in the clinical context, becoming an integral part of routine research and medical care, as well as a legal requirement in many national legislations (Hoeyer 2009). However, the process of adopting the requirements for informed consent in different contexts has not all been smooth sailing. As a consequence of the growing complexity of medical procedures and knowledge about the associated risks and implications, informed consent documents have often become lengthy and difficult to understand (Manson and O’Neill 2007). Reaching the standards of explicit, specific and simultaneously truly *informed* consent may be often very difficult to achieve—a topic which has been widely debated in academic literature (Manson and O’Neill 2007). Many studies have reported low levels of readability and/or understandability of informed consent forms in the USA, which is particularly worrisome given the prevalence of low levels of (health) literacy in the population (Sugarman et al. 1999; Paasche-Orlow et al. 2004). Furthermore, the importance of providing legible informed consent documents has been supported by medical malpractice case law (Paasche-Orlow 2005). Importantly, recognition of the relevance of patients’ perspectives and needs, as well as the provision of adequate information by a physician, has given rise to concepts and practices such as shared decision making (i.e. between physician and patient), patient-centred care and reasonable-patient informed consent standards, which have been implemented in the US and UK healthcare practice (Krumholz 2010; Spatz et al. 2016). While these approaches stress the role of communication processes between a physician and patient, they do not diminish the importance of providing written documents, which should facilitate the discussion, and can be taken home by a patient in order to be considered and reflected upon at the patient’s own pace (Krumholz 2010). Therefore, adequate readability and comprehensibility of informed consent forms remain vital elements of the informed consent process.

### Informed consent in genetics and genomics

Genetics is a relatively advanced subset of biology, and the task of successfully communicating genetic concepts to a public unfamiliar with the subject can be challenging (McBride et al. 2010). Explaining issues related to genomics, including the use of next-generation sequencing in order to perform whole-exome and whole-genome sequencing (WES, WGS), adds to this complexity. These approaches generate an unprecedented amount of information, potentially about thousands of phenotypes, including diseases that may also hold relevance for family members of probands. In addition, the interpretation of these findings may change with time (Pinxten and Howard 2014). Whole-genome and whole-exome sequencing are being increasingly used in research, clinical and direct-to-consumer settings, and their use is predicted to expand (Rehm 2017). A number of recommendations for informed consent for WGS have been issued to address this challenge. These documents outline and discuss the elements that should be included in the informed consent process and often emphasize the crucial role of pre-test counselling (Presidential Commission for the Study of Bioethical Issues 2012; van El et al. 2013; ACMG Board of Directors 2013; Ayuso et al. 2013).

A few studies analysed the content of IC forms for WGS and/or WES and discussed the presence (or absence) of a list of core elements (Jamal et al. 2013; Henderson et al. 2014; Niemiec et al. 2016). Two of these studies also report on readability of IC forms (Jamal et al. 2013; Henderson et al. 2014). Henderson and co-authors analysed nine informed consent forms for WES and WGS studies funded by the US National Human Genome Research Institute and National Cancer Institute. Readability was evaluated by the Flesch-Kincaid formula giving a median of 10.8 grade level, which indicates that after 10.8 years of education, an average student would understand most of the text present in the forms (Henderson et al. 2014). Jamal et al. (2013) analysed six informed consent forms provided by US-based laboratories offering clinical exome sequencing. The median readability score (Flesch Reading Ease) among documents was 40 (corresponding to between high-school and some college grade levels) (Flesch 1949; Jamal et al. 2013). Both of these studies indicate that the readability grade level is above the average recommended grade level of 8 for IC forms as stated by Institutional Review Boards of US medical schools (Paasche-Orlow et al. 2003). These results suggest that even if the forms include the required elements of information, they may not be comprehensible to many patients since almost half of Americans read at or below grade level of 8 (Paasche-Orlow et al. 2003).

Given the particular challenges of communicating information about WGS and WES, their increasing use in health care and the importance of providing the information in a readable manner, we aimed to provide additional insights into the readability level of a larger sample of informed consent forms for WGS and WES in the clinical context using two readability tests.

## Methods

### Search and inclusion criteria for IC forms

The authors searched for informed consent forms using Google search engine (www.google.com) applying 12 combinations of terms from the following groups: (“informed consent”, “consent document”, “consent form”) and (“whole genome sequencing”, “whole exome sequencing”, “next generation sequencing”, “genome wide sequencing”). The search was performed between March and April 2016. Two pairs of authors independently conducted the search using the above search terms combinations. One hundred links retrieved in each search-term-combination were accessed and reviewed. Documents meeting the criteria of consent forms for clinical WGS/WES in English were included in this study. Consent forms developed primarily for research projects and forms that did not have a space for the patient’s signature were excluded. Additional consent forms that were not retrieved in the search, but that were known by the authors from other sources, were also included. The final collection of forms was read and studied for a number of different aspects, including information on return of results, use of samples and data in research, as well as readability. Herein, we present only the results of the readability study.

### Characteristics of the forms

The following information was extracted from the IC forms and/or websites of WGS/WES providers: name of provider; country of origin; type of provider (type 1: universities/hospitals/medical centres and their “in-house” and/or owned laboratories; type 2: laboratories/companies not related to a university/hospital/medical centre); for what type of test a form is used (WES or WGS or both); and who can be tested (child, adult). This information was obtained independently by two authors and discrepancies were resolved in discussion.

### Readability

#### Preparation for analysis

The forms were prepared for the readability analysis by directly converting files from an original portable document format (pdf) to a docx file format or by copying and pasting information from the original document into a Word docx file. Final versions of converted or copied files were verified for accuracy with the original file, and any discrepancies were corrected. Additional sections included in the original files with the informed consent forms were excluded for this analysis (e.g. requisition forms, tables for patient information, sample information, address, payment options, clinical information, physician’s statements, text explicitly aimed at physicians). Sections of forms addressed to family members submitting a sample for validation of patient’s results were included. Headings were also included and each was treated as a complete sentence, even when there was no period in the end. The following phrases and words not constituting the main part of the informed consent form text were removed so that the program would not treat them as full sentences and consequently conflate the resulting readability scores: address and contact information of a provider; indications of fields for signatures, initials, names, addresses and dates of birth; dates of updating/creating forms; pages numbers. Website addresses found anywhere in the text were also removed. Numerals were fully syllabized (i.e. sounded out) in the tests used.

#### Readability measures

A number of different readability tests have been developed for evaluating reading grade levels. These are based on evaluating parameters, such as sentence length and the number of syllables in words. The reported grade level indicates the number of years of education that a person must have completed to understand the text assessed. In this study, two tests were used to assess the readability: the SMOG^1^ formula developed by McLaughlin (1969) and the Flesch-Kincaid formula (McLaughlin 1969; Kincaid et al. 1975). Basic characteristic of the formulas is shown in Table 1. The Flesch-Kincaid formula is the most commonly used for analysis in recent health care literature (years 2005–2008), which is likely to be the result of the embedding of this formula in Microsoft Word software (Wang et al. 2013). However, the Flesch-Kincaid formula is expected to predict only about 75% of comprehension (when validated on multiple-choice test), meaning that a person who completed the grade level indicated in the test would be able to comprehend approximately 75% of the text (Kincaid et al. 1975). Distinctively, the SMOG formula was developed to predict 100% comprehension (validated using McCall-Crabbs Standard Test Lessons in Reading based on multiple choice tests) (McLaughlin 1969). For this reason, the SMOG appears to be a more adequate test to evaluate informed consent forms for which 100% comprehension is expected (Wang et al. 2013). Hence, we used the SMOG test as the main evaluative calculation, although we also employed the Flesch-Kincaid formula to obtain results comparable to other studies using this test. Calculation of readability for the two groups of IC forms (type 1 and type 2, Table 3) was conducted using the SMOG test. The results obtained for these two groups were compared using Mann-Whitney statistical test.

**Table 1:** Information regarding the readability formulas used to analyse consent forms

	Flesch-Kincaid formula	SMOG^a^ formula
Original development and reference	The formula has been designed for evaluating readability of technical texts for US military by Kincaid (Kincaid et al. 1975).	McLaughlin (McLaughlin 1969)
Analysis based on	Sentence length and syllable count	Number of complex words (3 and more syllables)
Easier formula for manual calculation (not used in this study)	G = (12*(B/W)) + (0 .4*(W/S)) – 16 G—grade level W—number of words B—number of syllables S—number of sentences	G = FLOOR(√C) + 3 G—grade level C—number of complex words (3 and more syllables) FLOOR—round the result of (√C) down to the closest perfect square.
Higher precision formula used by the software in this study	G = (11.8*(B/W)) + (0.39*(W/S)) − 15.59	G = 1.0430*√C + 3.1291

^a^Originally, McLaughlin recommended using 10 consecutive sentences from the beginning of the text, 10 sentences from the middle and 10 from the end; the formula was meant to facilitate manual calculations. In our study, the calculations were based on the whole text (and not subsamples of the text) and standardized

Both tests were performed using the software Readability Studio Professional Edition for Windows, version 2015 (Oleander Software Ltd., Vandalia, Ohio). The calculations were based on the whole text (and not subsamples of the text) and standardized if needed. Additionally, we calculated the word count of informed consent documents as a rough indicator of the time required to read the text.

### Information about the informed consent process

In order to have some insight into the informed consent process, we also report on the presence of statements mentioning pre-test counselling as well as the sections of the forms aimed directly at health care professionals (HCPs).

## Results

### Characteristics of forms

We identified 36 informed consent forms for clinical WGS/WES in English: 32 forms were retrieved through the Google search; 4 forms were identified from WES/WGS providers with which the authors were familiar. The majority of forms come from various types of providers in the USA, are used for WES and are targeted at both adult and children patients. The complete list of form characteristics is outlined in Table 2.

**Table 2:** Information about IC forms: the country of origin, provider, type of test, groups to which it is offered

Characteristics	Number of forms
Total number of forms	36
Country of origin	
USA	29
Germany	2
The Netherlands	2
Australia	1
Canada and Germany	1
Finland	1
Provider	
Type 1: university/hospital/medical centre and their “in-house” and/or owned laboratories	18
Type 2: company/laboratory not related to a university/hospital/medical centre	18
Type of test	
WGS	5
WES	24
WGS and WES	4
WGS, WES and another genetic test	3
Target group	
Only adults	3
Only children	1
Adults and children	30
Not specified	2

### Readability results

**Fig. 1:**
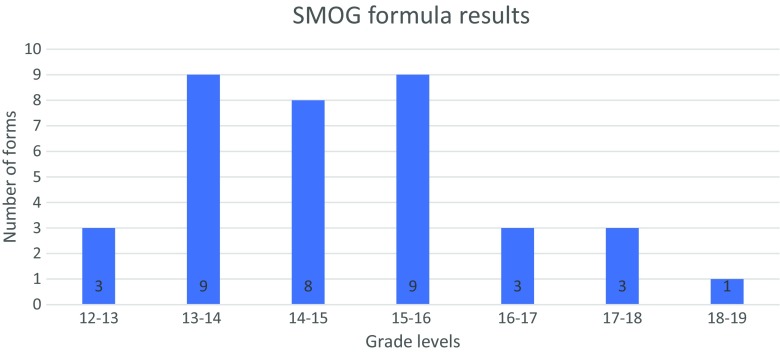
Results of the SMOG calculation for all the forms studied. The indicated ranges include the scores that are equal to or greater than the lowest bound and less than the largest bound for the range

**Fig. 2:**
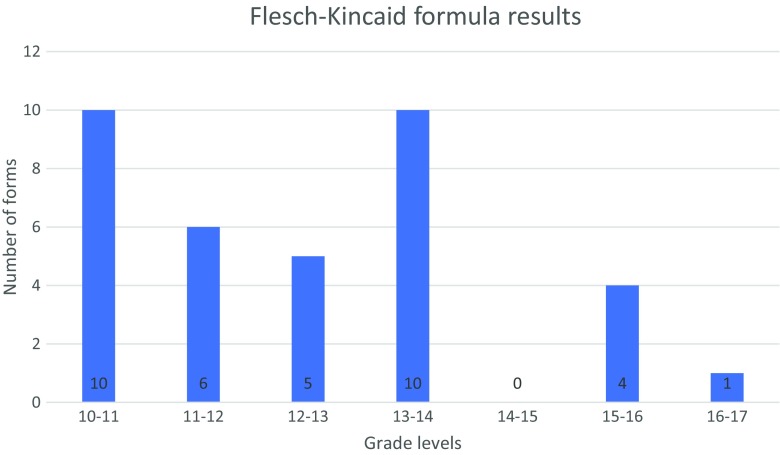
Results of the Flesch-Kincaid calculation for all the forms studied. The indicated ranges include the scores that are equal or greater than the lowest bound and less than the largest bound for the range

**Fig. 3:**
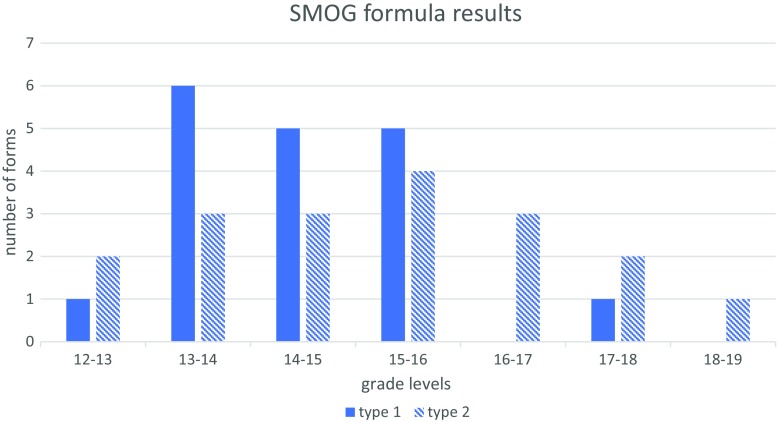
Comparison of readability between groups of IC forms using the SMOG formula. Type 1: universities/hospitals/medical centres and their “in-house” and/or owned laboratories; type 2: laboratories/companies not associated with a university/hospital/medical centre. The indicated ranges include the scores that are equal or greater than the lowest bound and less than the largest bound for the range

Figures 1 and 2 illustrate the results of the SMOG and the Flesch-Kincaid formulas. The range of grade level scores for the SMOG formula was 12.7–18.4, with a mean grade level of 14.8 and median of 14.75. For Flesch-Kincaid, the range was 10.3–16.4; mean 12.5 and median of 12.2. The word count ranged between 204 and 3017 words, with a mean of 1679 words and median of 1489. Figure 3 and Table 3 include the values for the SMOG formula and word count obtained in two groups of IC forms: universities/hospitals/medical centres and their “in-house” and/or owned laboratories (type 1) and laboratories/companies not associated with a university/hospital/medical centre (type 2). No significant differences were found between the two groups with respect to word count or readability grade levels.

**Table 3:** Grade levels obtained for two categories of IC forms. The Maan-Whitney test was used for comparison of results between these two groups of test providers

	Type 1: universities/hospitals/medical centres and their “in-house” and/or owned laboratories	Type 2: laboratories/companies not associated with a university/hospital/medical centre	*p* value and Z-score
Grade level SMOG	Range 12.9–17; median 14.5	Range 12.7–18.4 Median 15.4	Z = 1.61 *p =* 0.1
Word count	Range 204–3017; median 1405	Range 544–2785; median 1541	Z = 0.17 *p* = 0.85

### Information about informed consent process

Thirty-two of the forms mentioned some form of pre-test genetic counselling outlining, for example, that patients should consider, seek and/or obtain pre-test genetic counselling, or that pre-test genetic counselling is recommended/required. Twenty-one forms included text aimed at a HCP stating that a HCP has provided/discussed relevant IC information and/or offered/ensured providing of pre-test counselling.

## Discussion

### Very low readability of IC forms

All of the 36 forms studied have a higher reading grade level than that recommended (by US medical schools Institutional Review Boards) for IC forms, which is, on average, a grade level of 8 (Paasche-Orlow et al. 2003). The values obtained in the SMOG calculation are higher than those from the Flesch-Kincaid. This result is expected as the SMOG formula aims to predict 100% comprehension, while the Flesch-Kincaid formula would predict only about 75% comprehension (when validated on multiple choice test) (McLaughlin 1969; Kincaid et al. 1975). Our results correspond with the relatively high reading grade levels of informed consent forms obtained by Jamal et al. (2013) and Henderson et al. (2014), which indicated the median grade level of high-school to some college in the Flesch Reading Ease formula, and median of 10.8 grade level with the Flesch-Kincaid formula, respectively (Flesch 1949; Jamal et al. 2013; Henderson et al. 2014). The word count of the IC forms we studied ranged from 204 to 3017 words, with a mean of 1679 words and median of 1489, suggesting that a person would need, at least, between 1 and 15 min to read the informed consent form content aimed at patients (assuming the pace of reading of 200 words per minute) (Bell 2001). However, given the fact that the readability of the texts studied is low, an average patient would probably need much more time to assimilate the content of an IC form. These findings are in line with those of Jamal et al. (2013), which indicate the median word count among the six studied IC forms for WES is 1154, and the range is 724 to 3429 words (Jamal et al. 2013). Both the results herein and Jamal et al.’s word count results are lower than the values obtained by Henderson et al. (2014) in a study of 9 IC forms for WES/WGS (mean = 4588 words, range 2917–5757 words) (Jamal et al. 2013; Henderson et al. 2014). This difference may be related to the fact that Henderson et al. (2014) analysed consent forms used in a research context, and these may have contained additional information such as about the study design (Henderson et al. 2014).

The results indicating low readability of IC forms are not surprising, particularly when comparing them to studies of IC forms in the context of other medical procedures (Sugarman et al. 1999). However, it is interesting that *none* of the forms in this study, or other previous studies investigating IC for WGS, reaches the average recommended readability level of 8th grade (Henderson et al. 2014; Jamal et al. 2013). This indicates that IC forms may fail to fulfil their intended function of providing understandable information to patients and facilitating communication. The high scores obtained in the SMOG and Flesch-Kincaid formulas indicate that the documents studied use many complex, long words, which may often be technical and therefore difficult to understand to an average reader. Indeed, some sections of IC form text were difficult to understand even for the authors; one could imagine that it would be even more complicated for a person not familiar with vocabulary used in genetics, for instance:

*Diagnostic findings not related to phenotype in childhood onset conditions—a single pathogenic or likely pathogenic variant in genes that are known to cause autosomal dominant or X-linked childhood onset conditions, as well as two pathogenic or likely pathogenic variants in genes that are known to cause autosomal recessive childhood onset conditions, even if they are unrelated to the patient's phenotype, will be reported.* (IC form number 18. The length of this sentence is 64 words; the score in the SMOG formula is 19).

This lack of adequate provision of information in IC forms appears particularly worrisome given that some of the companies offering WES/WGS included in this study also advertise the tests directly to consumers. In the direct-to-consumer advertising context, consumers may be provided with encouraging information about the benefits of the testing on the companies’ websites, and unless explained in the IC process, they may not be aware of all the limitations and risks of the testing (Singleton et al. 2012). The need for legible IC forms seems to be even more relevant when WGS and WES is offered to minors; if possible, consent or assent should be obtained from children when testing is offered (American Academy of Paediatrics 2013). Therefore, clear and informative content of IC forms can be very valuable in this context.

Since we hypothesized that the potentially greater presence and involvement of HCPs in designing IC forms might result in increased readability of the forms, we assigned the IC forms to two different groups, assuming that the involvement of HCPs is higher in the first group: group 1—university/hospital/medical centres and their "in-house" and/or owned laboratories; group 2—companies/laboratories not associated with a medical centre/hospital/university. Readability and word count was compared among these groups (Table 3 and Fig. 3). No statistically significant differences were found between these two IC form types with regard to readability scores and word count. These results suggest that involvement of health care professionals/genetic counsellors with experience in communication may be similar in these two groups. Indeed, the recent data indicate that an increasing number of genetic counsellors work in diagnostic laboratories (Waltman et al. 2016). The process of designing informed consent forms, including the involvement and roles of various experts may be worth investigating further.

### Role of a HCP in the informed consent process

The requirement or suggestion to undergo pre-test counselling present in many forms studied, as well as the sections of text stating that a HCP has provided relevant information to the patient (which often should be signed by a HCP) seem to place an obligation on HCPs and genetic counsellors. These statements imply that the physician is responsible for ensuring that the patient is adequately informed and understands the information provided, even if the consent form is not easy to comprehend. Consequently, given the low readability of the forms and the stated obligation of a HCP to explain the relevant information, IC forms in this context may take a role of a “checklist” for a HCP indicating which elements (s)he should explain to a patient, rather than being a sole explanatory material for a patient. Indeed, a study by Bernhardt et al. (2015) showed that during pre-test counselling sessions for genomic sequencing, genetic counsellors and research coordinators modified and adjusted (depending on the context) the information provided to the patients from that presented in the IC forms (Bernhardt et al. 2015). Moreover, the study reported that genetic counsellors and research coordinators “*recognized that most patients and participants cannot attend to, let alone understand, all of the information contained in the consent documents*” (Bernhardt et al. 2015). Undoubtedly, the HCP’s role (and often obligation) to communicate and provide information is vital for the IC process, not only for genomic testing but in the context of all clinical procedures or tests requiring informed consent. However, considering the predictions that genomics is likely to become part of mainstream practice in medicine, WGS and WES may be increasingly offered by primary care professionals who may not yet have sufficient training or experience to be able to communicate effectively with patients about genomics (Christensen et al. 2016). In such cases, primary care professionals may be more dependent on IC forms as a communication tool to explain WGS/WES to patients. Consequently, in these circumstances, the explanatory and educational role of informed consent forms should not be underestimated.

The appropriate means of communicating about genomics in IC forms (e.g. usage of understandable vocabulary, length of document etc.) need to be explored, implemented, monitored and revised as needed. To obtain more comprehensive evaluation of the functionality of informed consent forms, additional methods, such as Suitability Assessment of Materials could be applied (Kloza et al. 2015). Furthermore, insights from health professionals who have experience in obtaining informed consent for genomic testing could help improve the quality of informed consent forms. For example, the issues indicated by genetic counsellors as most important for patients and most likely to be misunderstood could gain more attention when designing informed consent forms. In addition, reducing the length of other (potentially less relevant to informed consent) sections of IC forms such as descriptions of the technical aspects of sequencing might increase the readability of the forms (Bernhardt et al. 2015). Furthermore, investigating patients’ needs and understanding when communicating about genomics could be another important element in the effort to design adequate informed consent information (Parry and Middleton 2017).

### Limitations

The limitations of this study include, firstly, that the consent forms were collected at one given point of time, in one language (English) using a specific strategy aimed at finding documents available online. We acknowledge that we may have missed some documents that are currently in use but not publicly available online, and that the studied forms we found may no longer be in use. The study of additional forms in other languages than English could also be of value. Secondly, there are limitations inherent to the readability formulas used. For example, not all the (potentially) difficult words have more than two syllables (for instance “genome”). Furthermore, the readability formulas do not evaluate all the elements influencing readability, for example, graphic design, font type and size and document layout. Finally, readability and comprehension are distinctive measures. However, the SMOG and Flesch-Kincaid formulas were validated in tests aiming at evaluating comprehensibility; it has been questioned whether some of them accurately reflect comprehension (Wang et al. 2013). Therefore, the readability results only provide an estimation of comprehensibility of informed consent forms. In order to evaluate factual understanding of the documents, a study surveying patients should be conducted.

## Conclusions

Based on the 36 IC forms identified, our results suggest that the IC forms for use in WES/WGS in the clinic may not adequately fulfil their function of explaining relevant information to patients. This function seems to be transferred to some extent to genetic counsellors and/or health care professionals, which may be problematic if a HCP does not have sufficient training in genomics to be able to explain the information to patients. Therefore, moving forward, along with educating HCPs in genomics, it will be essential for good examples of informed consent forms to be developed that will communicate relevant information effectively and facilitate the process of informed consent. Engaging expert groups including clinical geneticists, genetic counsellors, communication professionals and patients may facilitate this task. In order to ensure responsible implementation of genomic technologies, securing an adequate, truly informed consent process should be taken seriously and adequate resources should be allocated to enable fulfilling this task.

## Footnotes

1 SMOG is often referred to as Simple Measure of Gobbledygook in the literature; however, in the author’s intention, it is a reference to another readability grading formula (Gunning’s Fog Index (Gunning 1968)): “*I call my system of readability prediction SMOG Grading in tribute to Gunning’s Fog Index* (*The term also refers to my birthplace, smog having first appeared in London, though, like so many other things, it has since been improved upon in several American cities*)”. (McLaughlin 1969)
